# Supplementary material for: SwarmMAP: swarm learning for decentralized cell type annotation in single cell sequencing data
Source: NPJ Syst Biol Appl. 2026 Feb 18;12:41. doi: 10.1038/s41540-026-00667-6 (PMC13031268; doi:10.1038/s41540-026-00667-6)
Supplement: Supplementary file 1 — Supplementary information [file 41540_2026_667_MOESM1_ESM.pdf]

Supplementary Information for the paper: "SwarmMAP: Swarm Learning for Decentralized Cell Type Annotation in Single Cell Sequencing Data"

Oliver Lester Saldanha[1†], Vivien Goepp[4†], Kevin Pfeiffer[1], Hyojin Kim[4], Jie Fu Zhu[1], Rafael Kramann[4], Sikander Hayat[4, 5, 6*], Jakob Nikolas Kather[1,2,3*]

[1] Else Kroener Fresenius Center for Digital Health, Technical University Dresden, Fetscherstraße 74, Dresden, 01307, Saxony, Germany .

[2] Department of Medicine I, Faculty of Medicine and University Hospital Carl Gustav Carus, Technical University Dresden, Fetscherstraße 74, Dresden, 01307, Saxony, Germany .

[3] Medical Oncology, National Center for Tumor Diseases (NCT), University Hospital Heidelberg, Im Neuenheimer Feld 460, Heidelberg, 69120, Baden-Wuerttemberg, Germany .

[4] Department of Medicine 2, RWTH Aachen University, Medical Faculty, Pauwelsstrasse 30, Aachen, 52074, North Rhine-Westphalia, Germany .

[5] Cardiovascular Research Institute and Department of Medicine, Icahn School of Medicine at Mount Sinai, 1 Gustave L. Levy Place, New York, 10029-5674, NY, USA .

[6] Windreich Department of Artificial Intelligence and Human Health, Icahn School of Medicine at Mount Sinai, 1 Gustave L. Levy Place, New York, 10029-5674, NY, USA .

*Corresponding author(s). E-mail(s): shayat@ukaachen.de; jakob_nikolas.kather@tu-dresden.de;
Contributing authors: oliverlestersaldanha25@gmail.com; vgoepp@ukaachen.de; kevin.pfeiffer@tu-dresden.de; genehaus@gmail.com; jeffzhu6969@gmail.com; rkramann@ukaachen.de;
†These authors contributed equally to this work.

Supplementary Figure 1: UMAPs of all datasets used in this study colored by cell type

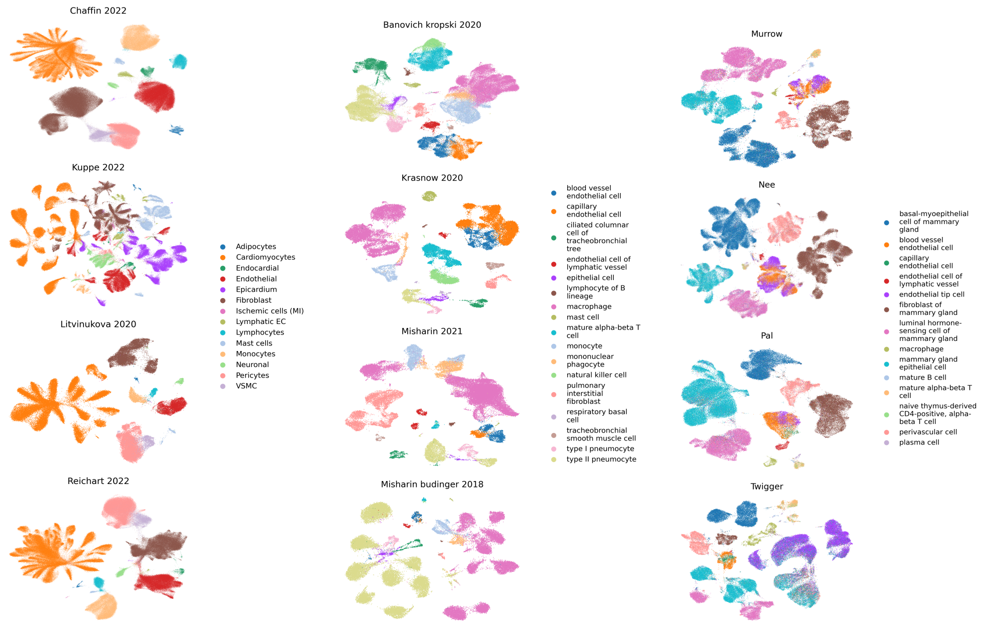

(a) Heart (b) Lung (c) Breast

Fig. 1: 2D Visualization of the dataset collections, colored by the manually-curated annotation of higher level ("cell types"). UMAP projections were computed independently for each study.

Supplementary Figure 2: UMAP of every dataset colored by cell subtype

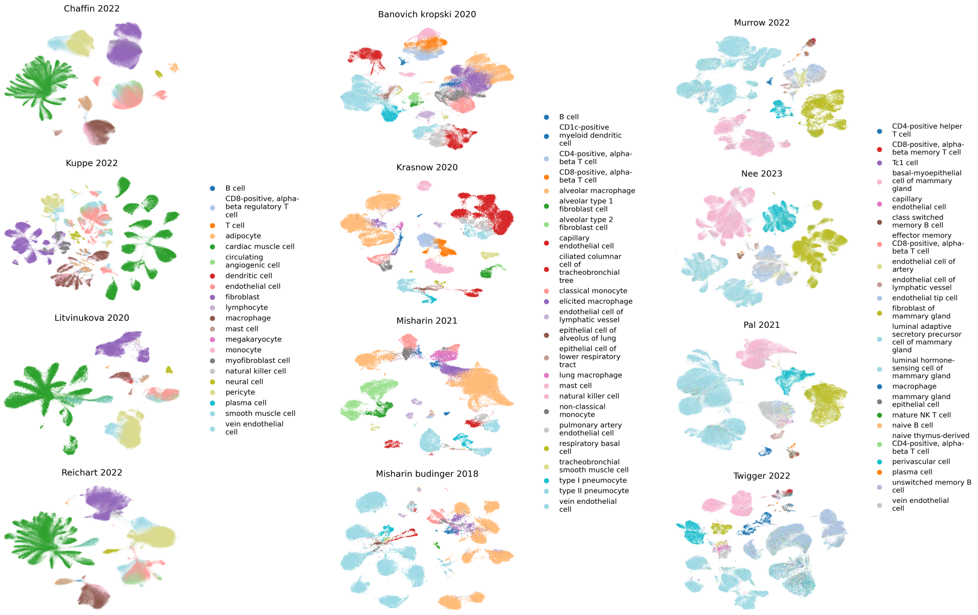

(a) Heart (b) Lung (c) Breast

Fig. 2: UMAP representation of the cell types in the heart, lung, and breast collections showing the cell subtypes.

Supplementary Figure 3: Comparison of MLP and XGBoost classifiers

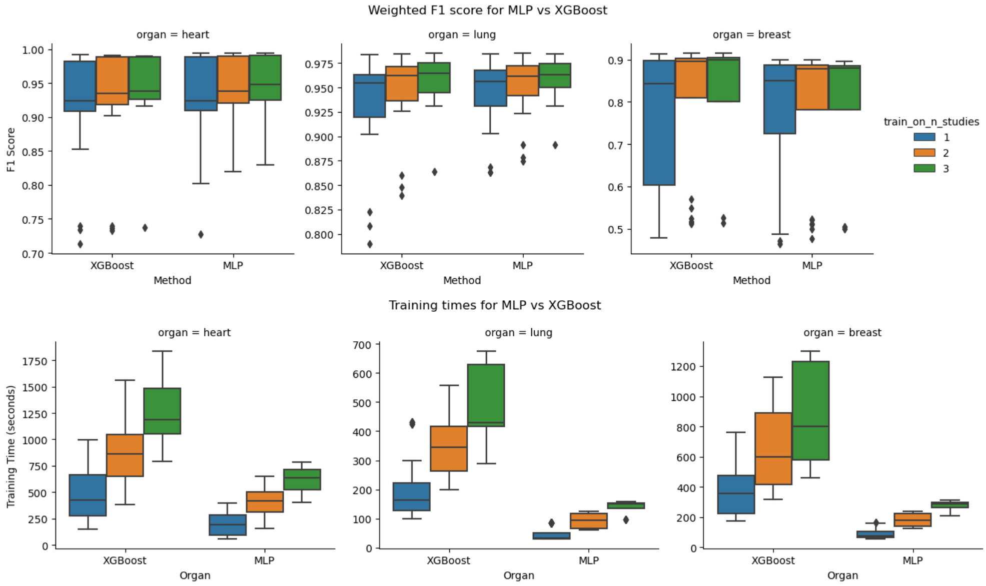

Fig. 3: Comparison of MLP and XGBoost classifiers in terms of weighted F1 score (top) and training time (bottom) at the cell type level in local learning.

Supplementary Figure 4: Weighted, macro, and micro F1 scores for local and swarm learning.

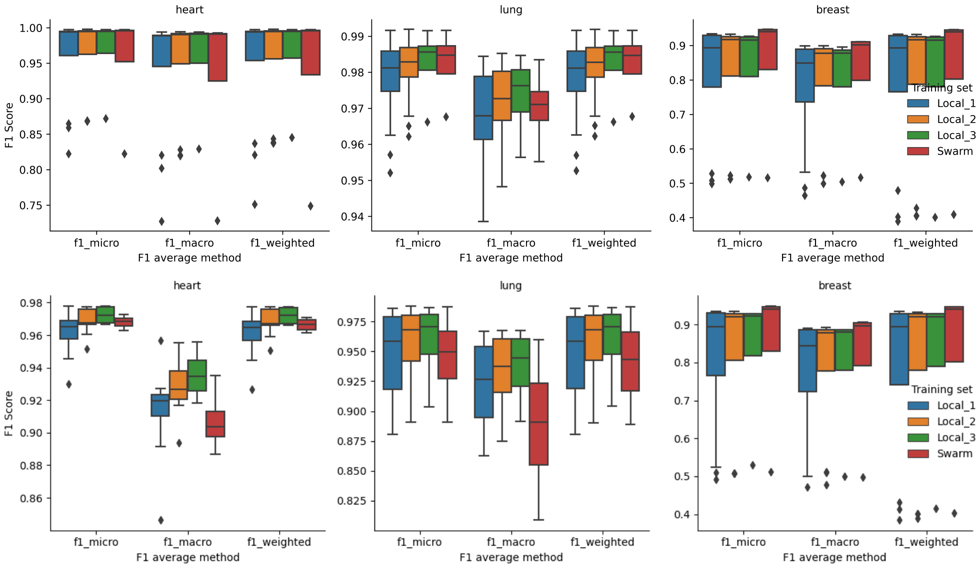

Fig. 4: Micro, macro, and weighted F1 scores for cell types (top) and cell subtypes (bottom) for LL and SL. Significance levels are not shown as no Mann-Whitney U tests are significant.

Supplementary Figure 5: Sankey plots between cell types and subtypes

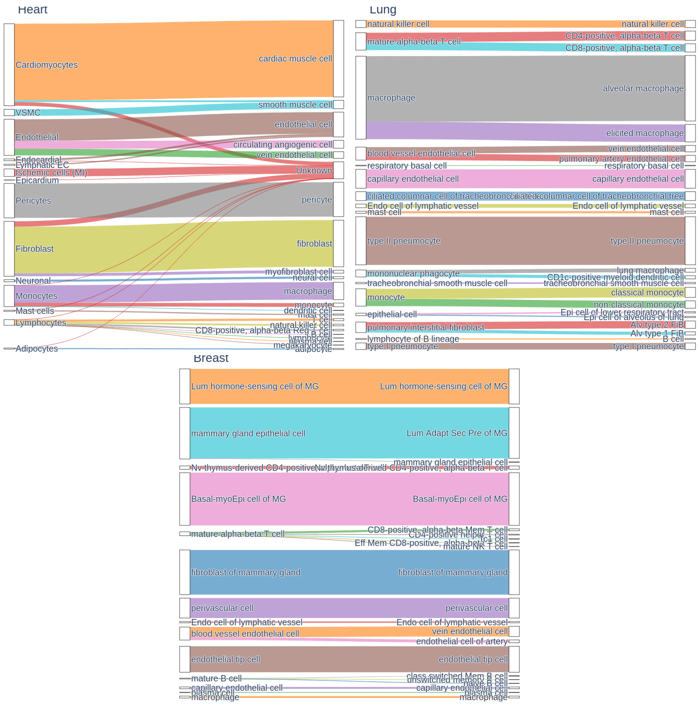

Fig. 5: Correspondence between cell types and subtypes for each organ.

Supplementary Figure 6: Confusion matrices for classifying cell subtypes

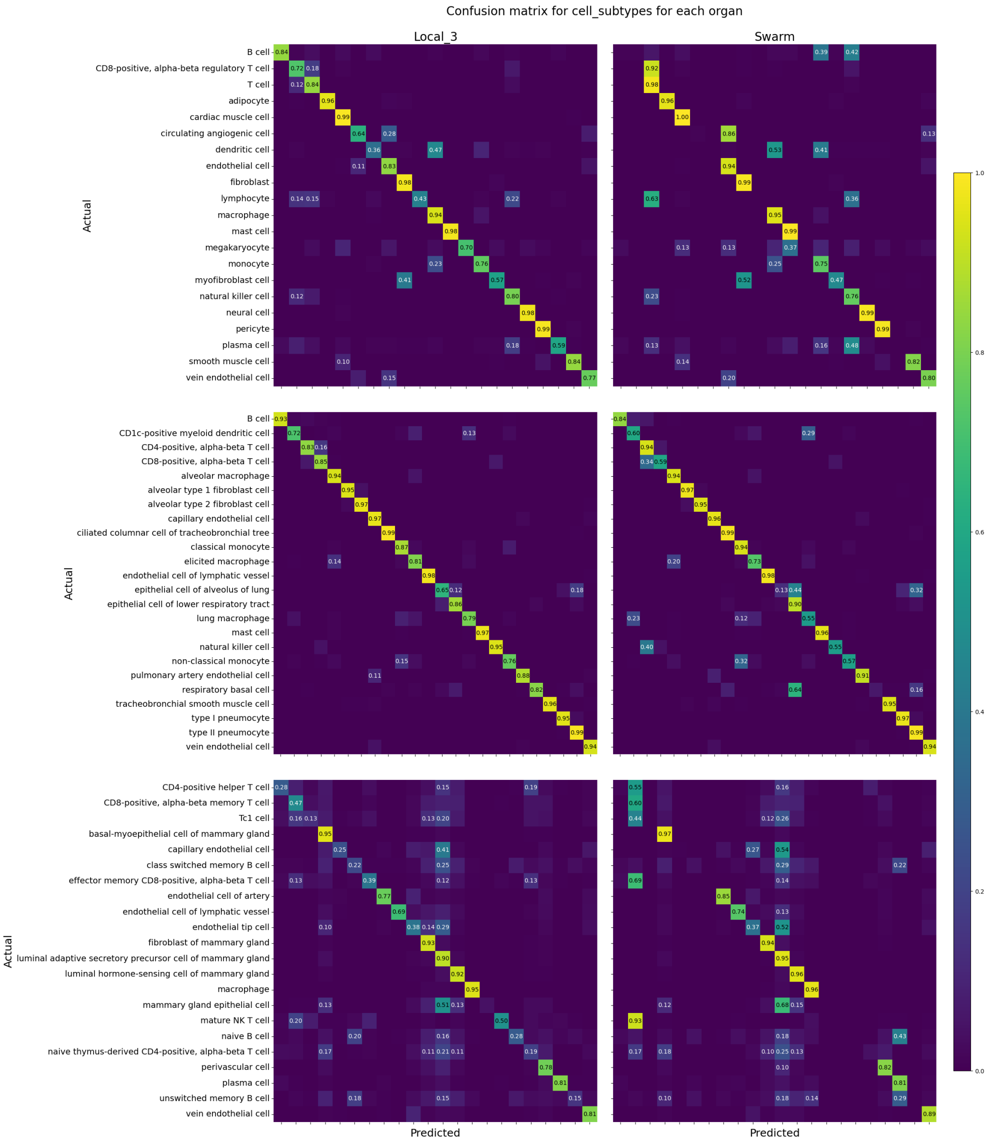

Fig. 6: Confusion matrices of Local_3 (left) versus Swarm (right) at cell subtype level for the heart (top), lung (center), and breast (bottom) datasets. The accuracies are averaged over all simulation runs and are normalized by row.

Supplementary Figure 7: Dendrograms of cell labels

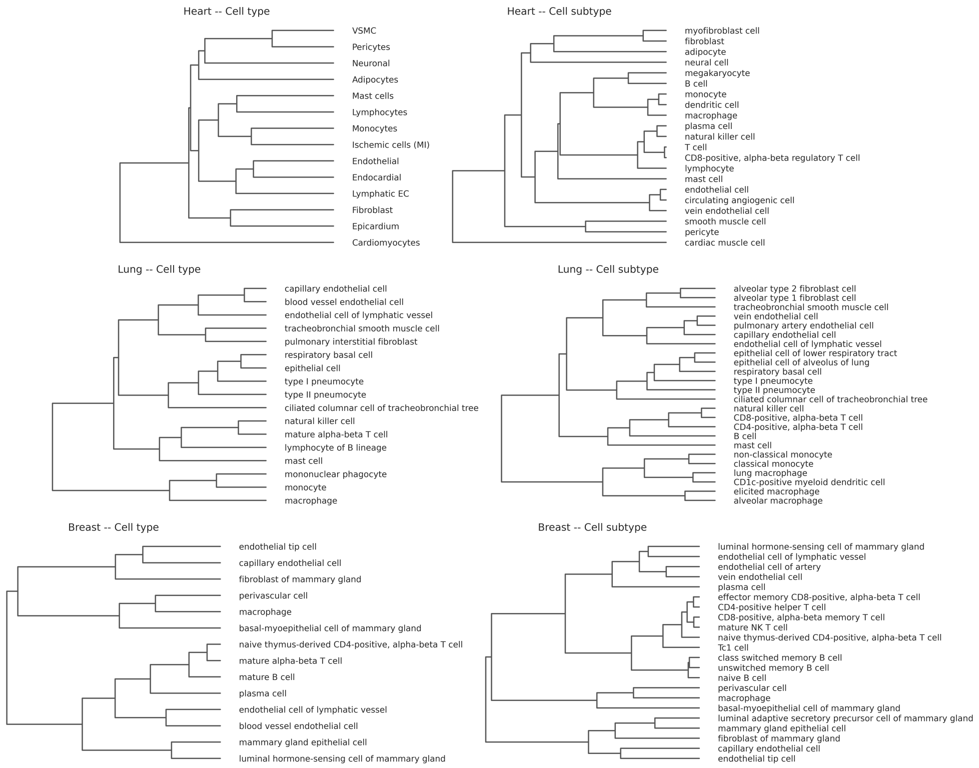

Fig. 7: Ontology of cell types and subtypes obtained by hierarchical clustering of collections after dataset integration.

Supplementary Figure 8: Cell type composition per dataset

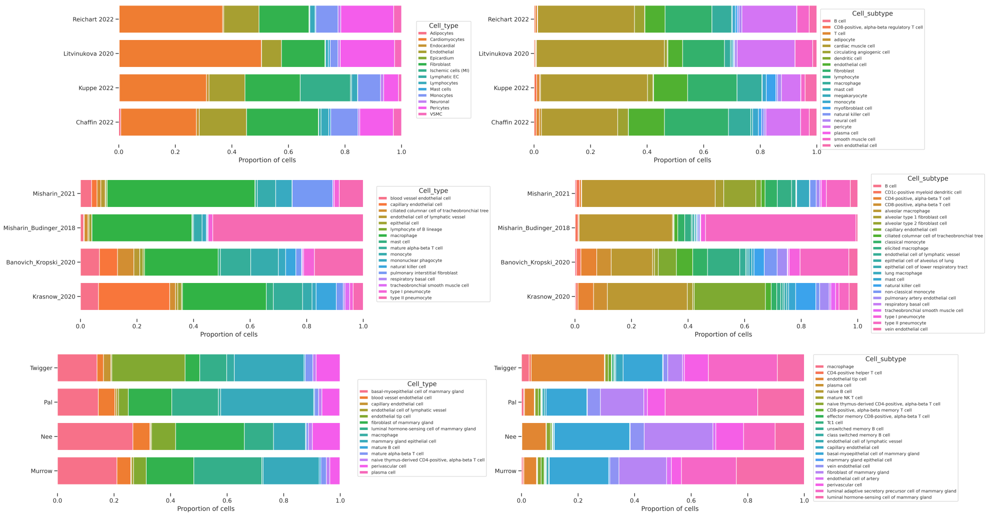

Fig. 8: Cell composition of all datasets, at both type and subtype levels.

Supplementary Figure 9: Effect of the number of HVGs

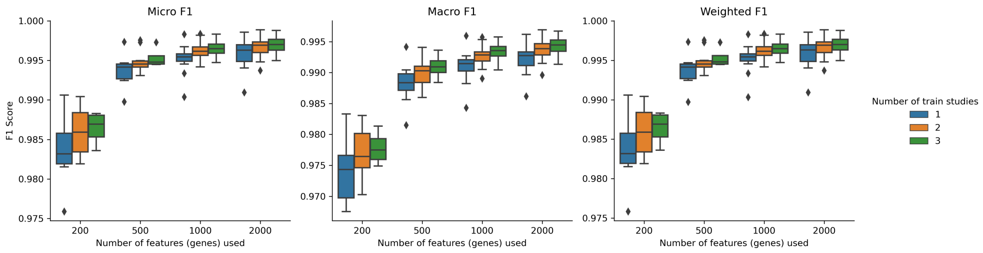

(a) Heart

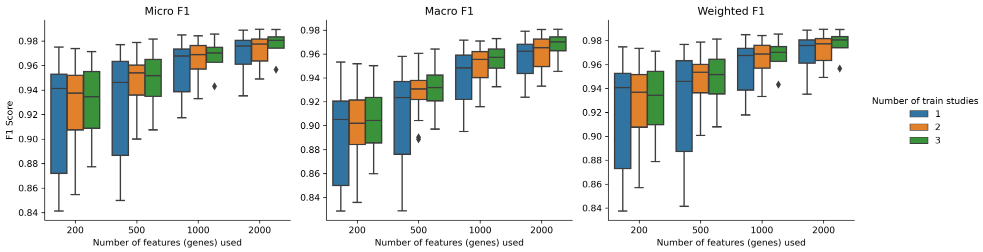

(b) Lung

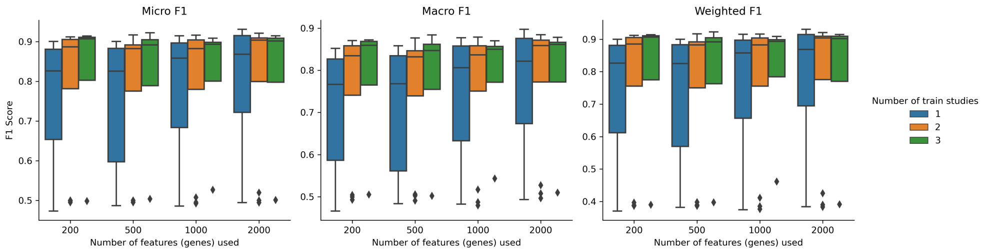

(c) Breast

Fig. 9: Classification performance for various numbers of HVGs selected at preprocessing.

Supplementary Figure 10: Effect of using a low-dimensional embedding

Our classifier learns cell type compositions in the presence of batch effects between datasets without needing to account for these batch effects. This is possible because the feature space is the (normalized) counts. If a low-dimensional embedding were used instead, the classifier could not recover batch-effect agnostic decision boundaries. This is illustrated by the classification performance of the MLP classifier trained on (i) the normalized counts, (ii) the PCA of the counts using 50 PCs, and (iii) the embedding provided by scVI (Figure 10). The classification performance is significantly lower when using the low-dimensional embeddings, across organs and F1 scores average methods, with an even lower performance for the scVI embedding. This suggests that in the presence of batch effects, the low-dimensional embeddings do not capture the relevant information for cell type classification, and that the classifier is not able to recover the cell type composition in the presence of batch effects when using these embeddings.

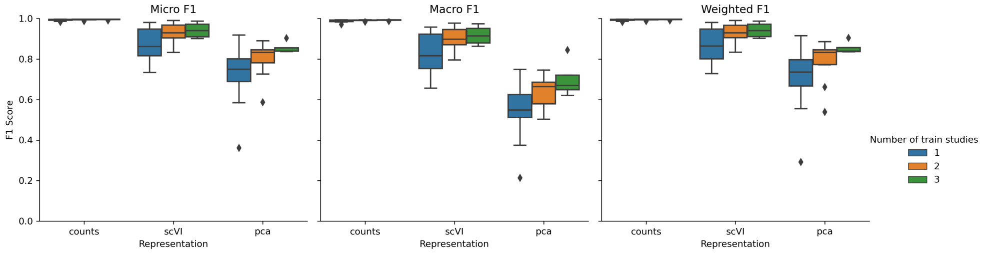

(a) Heart

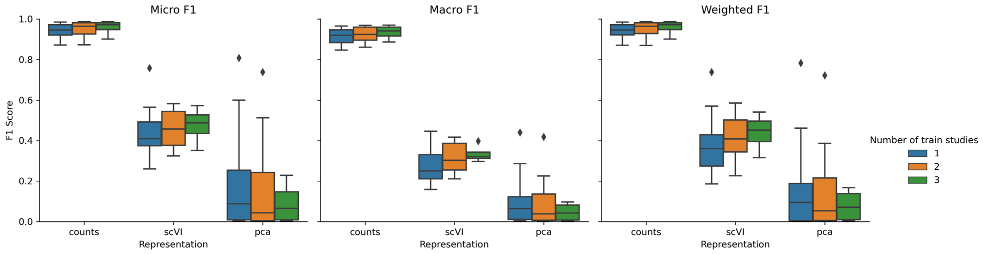

(b) Lung

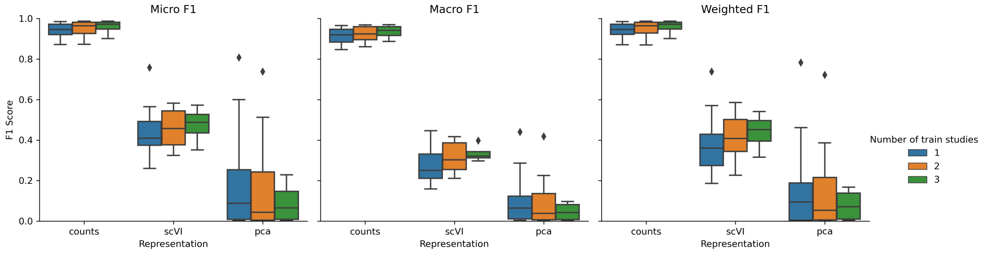

(c) Breast

Fig. 10: Classification performance using different representations of the data.

Supplementary Figure 11: Local learning F1 scores per cell subtype

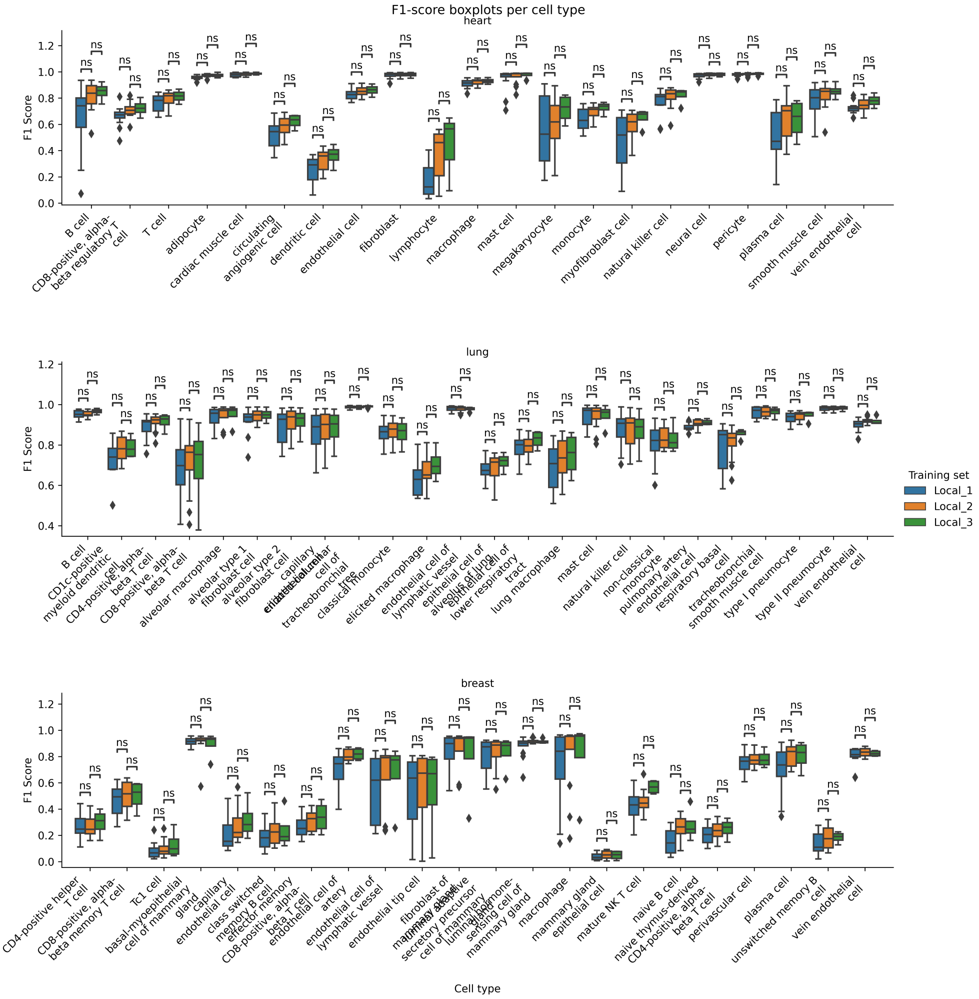

Fig. 11: F1 score for each cell subtype with LL when training on an increasing number of studies.

Supplementary Figure 12: Visualizing classification prediction score

Both local and Swarm classifiers display heterogeneous classification performance across cell types. To understand the reasons thereof, Figure 12 compares the true label with its prediction score. The results are represented for the two best classified and two worst classified cell types, for every organ. The values are represented in the UMAP of the test study. On one experiment experiment from Local_3 is considered for each organ and the test studies used are "Litvinukova 2020", "Misharin 2021", and "Twigger 2022" for heart, lung, and breast collections, respectively.

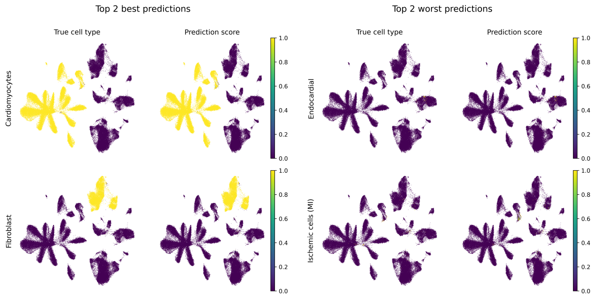

(a) Heart

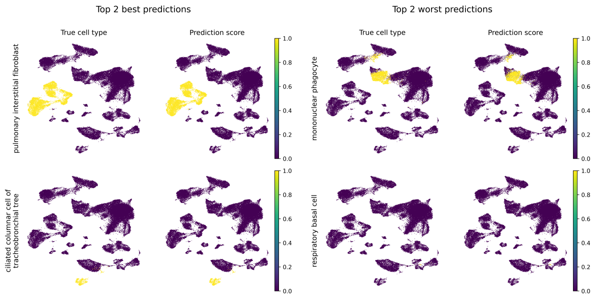

(b) Lung

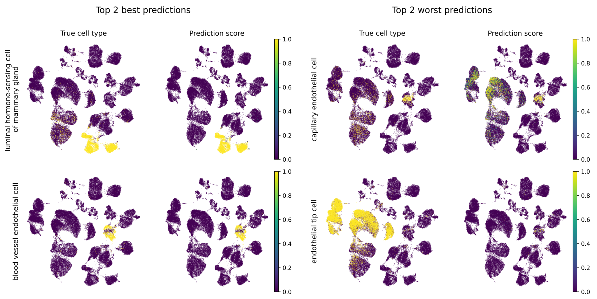

(c) Breast

Fig. 12: Top 2 best (left) and worst (right) classified cell types for lung. For each row, the left pane shows the true cell types and the right pane shows the prediction score.

Supplementary Figure 13: Detailed composition of datasets

Cell Type	Chaffin 2022	Kuppe 2022	Litvinukova 2020	Reichart 2022
Adipocytes	3914	457	150	599
Cardiomyocytes	149933	58279	42695	132252
Endocardial	5688	1686	46	1390
Endothelial	93673	24064	5994	45095
Epicardium	217	0	9	61
Fibroblast	142441	37012	13030	63816
Ischemic cells (MI)	531	33620	113	839
Lymphatic EC	4584	794	62	321
Lymphocytes	15361	3980	847	6567
Mast cells	3925	157	582	1113
Monocytes	53995	15244	2316	28295
Neuronal	3656	2061	688	3585
Pericytes	66530	9961	16334	67622
VSMC	16248	2034	2087	10094
Total	560696	189349	84953	361649

(a) Heart Data Cell Types

Cell Subtype	Chaffin 2022	Kuppe 2022	Litvinukova 2020	Reichart 2022
B cell	184	132	9	210
CD8-positive, alpha-beta regulatory T cell	3978	1269	277	1485
T cell	6138	1481	235	2250
adipocyte	3903	457	150	599
cardiac muscle cell	146191	55383	36159	119486
circulating angiogenic cell	20365	2840	795	12474
dendritic cell	231	287	218	345
endothelial cell	69080	17640	4156	24899
fibroblast	122773	25517	11814	57790
lymphocyte	19	36	2	42
macrophage	42596	12874	1699	24324
mast cell	3683	147	436	907
megakaryocyte	5	36	10	20
monocyte	11165	2083	399	3624
myofibroblast cell	5876	4952	120	2399
natural killer cell	4761	827	248	2162
neural cell	3656	2061	688	3585
pericyte	66530	9961	16334	67622
plasma cell	94	96	19	300
smooth muscle cell	16512	2203	4997	15108
vein endothelial cell	14476	6043	1151	9414
Total	542216	146325	79916	349045

(b) Heart Data Cell Subtypes

Cell Type	Banovich Kropski 2020	Krasnow 2020	Misharin 2021	Misharin Budinger 2018
blood vessel endothelial cell	7729	2546	1862	458
capillary endothelial cell	7368	10041	903	83
ciliated columnar cell of tracheobronchial tree	6674	821	646	533
endothelial cell of lymphatic vessel	2395	323	721	161
epithelial cell	1335	529	145	391
lymphocyte of B lineage	618	135	204	29
macrophage	30004	11829	24704	14342
mast cell	913	986	46	70
mature alpha-beta T cell	12347	4132	345	157
monocyte	11277	1335	3084	1224
mononuclear phagocyte	2951	577	2732	619
natural killer cell	4179	2845	29	27
pulmonary interstitial fibroblast	2343	979	6787	144
respiratory basal cell	262	245	11	50
tracheobronchial smooth muscle cell	534	670	240	29
type I pneumocyte	4435	480	831	675
type II pneumocyte	19885	1428	4011	21588
Total	115249	39901	47301	40580

(c) Lung Data Cell Types

Cell Subtype	Banovich Kropski 2020	Krasnow 2020	Misharin 2021	Misharin Budinger 2018
B cell	618	135	204	29
CD1c-positive myeloid dendritic cell	1809	302	591	410
CD4-positive, alpha-beta T cell	6455	2184	273	120
CD8-positive, alpha-beta T cell	5892	1948	72	37
alveolar macrophage	16841	11279	22322	13403
alveolar type 1 fibroblast cell	826	766	1424	121
alveolar type 2 fibroblast cell	1517	213	5363	23
capillary endothelial cell	7368	10041	903	83
ciliated columnar cell of tracheobronchial tree	6674	821	646	533
classical monocyte	6026	788	2038	992
elicited macrophage	13163	550	2382	939
endothelial cell of lymphatic vessel	2395	323	721	161
epithelial cell of alveolus of lung	554	219	54	265
epithelial cell of lower respiratory tract	781	310	91	126
lung macrophage	1142	275	2141	209
mast cell	913	986	46	70
natural killer cell	4179	2845	29	27
non-classical monocyte	5251	547	1046	232
pulmonary artery endothelial cell	4103	1328	622	198
respiratory basal cell	262	245	11	50
tracheobronchial smooth muscle cell	534	670	240	29
type I pneumocyte	4435	480	831	675
type II pneumocyte	19885	1428	4011	21588
vein endothelial cell	3626	1218	1240	260
Total	115249	39901	47301	40580

(d) Lung Data Cell Subtypes

Cell Type	Murrow 2022	Nee 2023	Pal 2021	Twigger 2022
basal-myoepithelial cell of mammary gland	16967	58449	16793	13993
blood vessel endothelial cell	3961	13218	6816	2284
capillary endothelial cell	167	350	702	2603
endothelial cell of lymphatic vessel	620	867	822	360
endothelial tip cell	3617	18699	4039	25944
fibroblast of mammary gland	13592	53260	17882	5212
luminal hormone-sensing cell of mammary gland	19397	22624	19187	9551
macrophage	376	23	930	2696
mammary gland epithelial cell	16013	24790	38307	24660
mature B cell	464	475	379	438
mature alpha-beta T cell	1563	1736	1641	2717
naive thymus-derived CD4-positive, alpha-beta T cell	1015	3145	1364	1142
perivascular cell	2877	21482	7326	8485
plasma cell	97	121	244	83
Total	80726	219239	116432	100168

(e) Breast Data Cell Types

Cell Subtype	Murrow 2022	Nee 2023	Pal 2021	Twigger 2022
CD4-positive helper T cell	298	255	287	720
CD8-positive, alpha-beta memory T cell	848	1097	965	1063
Tc1 cell	201	247	285	641
basal-myoepithelial cell of mammary gland	16967	58449	16793	13993
capillary endothelial cell	167	350	702	2603
class switched memory B cell	211	228	257	285
effector memory CD8-positive, alpha-beta T cell	177	90	80	242
endothelial cell of artery	1341	2294	1466	672
endothelial cell of lymphatic vessel	620	867	822	360
endothelial tip cell	3617	18699	4039	25944
fibroblast of mammary gland	13592	53260	17882	5212
luminal adaptive secretory precursor cell of mammary gland	15692	24460	38109	24440
luminal hormone-sensing cell of mammary gland	19397	22624	19187	9551
macrophage	376	23	930	2696
mammary gland epithelial cell	321	330	198	220
mature NK T cell	39	47	24	51
naive B cell	149	159	50	98
naive thymus-derived CD4-positive, alpha-beta T cell	1015	3145	1364	1142
perivascular cell	2877	21482	7326	8485
plasma cell	97	121	244	83
unswitched memory B cell	104	88	72	55
vein endothelial cell	2620	10924	5350	1612
Total	80726	219239	116432	100168

(f) Breast Data Cell Subtypes

Fig. 13: Number of cell for each cell type and subtypes, for each organ.

Supplementary Table 1: Composition of datasets by cell and cell types

Collection	Organ	Study	Number of cells	Number of cell types	Number of cell subtypes
1	Heart [1]	Chaffin et al. [2]	560696 (542216)	14	21
		Kuppe et al. [3]	189349 (146325)	13	21
		Litviňuková et al. [4]	84953 (79916)	14	21
		Reichart et al. [5]	361649 (349045)	14	21
2	Lung [6]	Habermann et al. [7]	115249	17	24
		Travaglini et al. [8]	39901	17	24
		Grant et al. [9]	47301	17	24
		Misharin and Budinger [10]	40580	17	24
3	Breast [11]	Murrow et al. [12]	80726	14	22
		Nee et al. [13]	219239	14	22
		Pal et al. [14]	116432	14	22
		Twigger et al. [15]	100168	14	22

Table 1: Datasets used for training and testing SwarmMAP. Each data collection concerns one organ and is composed of 4 datasets, called "studies". For heart, some cells had unknown subtype labels and were filtered out when using subtypes. The corresponding sample sizes are provided in parentheses.

Supplementary Table 2: Number of donors in each dataset

Organ	Study	Number of donors
Heart	Chaffin 2022	42
	Kuppe 2022	20
	Litvinukova 2020	14
	Reichart 2022	68
Lung	Banovich Kropski 2020	38
	Krasnow 2020	3
	Misharin 2021	2
	Misharin Budinger 2018	8
Breast	Murrow 2022	28
	Nee 2023	22
	Pal 2021	21
	Twigger 2022	18
Total		284

Table 2: Number of donors for each organ and study.

Supplementary Table 3: Data download links

Collection	Organ	Dataset	Download link
1	Heart	Chaffin 2022	https://singlecell.broadinstitute.org/single_cell/study/SCP1303/
		Kuppe 2022	https://cellxgene.cziscience.com/collections/8191c283-0816-424b-9b61-c3e1d6258a77
		Litvinukova 2020	https://cellxgene.cziscience.com/collections/b52eb423-5d0d-4645-b217-e1c6d38b2e72v
		Reichart 2022	https://cellxgene.cziscience.com/collections/b52eb423-5d0d-4645-b217-e1c6d38b2e72e75342a8-0f3b-4ec5-8ee1-245a23e0f7cb
2	Lung	Banovich Kropski 2020<br>Krasnow 2020<br>Misharin 2021<br>Misharin and Budinger 2018	https://cellxgene.cziscience.com/collections/b52eb423-5d0d-4645-b217-e1c6d38b2e726f6d381a-7701-4781-935c-db10d30de293
3	Breast	Murrow 2022<br>Nee 2023<br>Pal 2021<br>Twigger 2022	https://cellxgene.cziscience.com/collections/b52eb423-5d0d-4645-b217-e1c6d38b2e7248259aa8-f168-4bf5-b797-af8e88da6637

Table 3: List of human heart, lung, and breast datasets used in this study. Download links of all datasets for CellxGene platform are provided.

Supplementary Table 4: Averages of weighted F1 scores for LL and SL.

Organ	Label	Training set	Weighted F1 score (Confidence Interval)
Heart	Cell type	Local_1	0.947 (0.896, 0.997)
		Local_2	0.957 (0.917, 0.996)
		Local_3	**0.958** (0.884, 1.032)
		Swarm	0.934 (0.813, 1.056)
Heart	Cell subtype	Local_1	0.961 (0.953, 0.968)
		Local_2	0.968 (0.964, 0.973)
		Local_3	**0.972** (0.966, 0.978)
		Swarm	0.966 (0.962, 0.971)
Lung	Cell type	Local_1	0.978 (0.97, 0.985)
		Local_2	0.981 (0.975, 0.987)
		Local_3	**0.982** (0.971, 0.993)
		Swarm	**0.982** (0.972, 0.992)
Lung	Cell subtype	Local_1	0.945 (0.922, 0.969)
		Local_2	0.954 (0.933, 0.975)
		Local_3	**0.958** (0.921, 0.995)
		Swarm	0.941 (0.899, 0.982)
Breast	Cell type	Local_1	0.786 (0.661, 0.91)
		Local_2	0.794 (0.664, 0.924)
		Local_3	0.79 (0.536, 1.044)
		Swarm	**0.809** (0.547, 1.07)
Breast	Cell subtype	Local_1	0.78 (0.653, 0.908)
		Local_2	0.791 (0.657, 0.926)
		Local_3	0.796 (0.547, 1.046)
		Swarm	**0.808** (0.544, 1.072)

Table 4: Averages of weighted F1 scores for LL and SL across all settings. Confidence intervals are computed using the t-distribution. For each setting, the highest value is highlighted in bold.

Supplementary Table 5: Runtime experiments

Label	Organ	Local_1	Local_2	Local_3	Swarm Learning
Cell type	Heart	211	420	631	2780
	Breast	90	179	272	1120
	Lung	45	92	138	610
Cell subtype	Heart	198	397	595	2687
	Breast	94	183	275	1114
	Lung	45	91	137	609

Table 5: Average runtimes of Local and Swarm learning (seconds).

References

[1] Bleckwehl, T., Schumacher, D., Heymanns, C., Maryam, S., Andries, A.-S., Jurgens, S.J., Amrute, J., Hoeft, K., Wu, X., Liu, Y., Shin, H., Milting, H., Sattler, S., Lavine, K., Nyberg, M., Bosteen, M., Pyke, C., Das, V., Baumgart, S.J., Kramann, R., Hayat, S.: Integrative single-cell and genetic profiling of human heart failure identifies targets for cardiomyocyte restoration. medRxiv (2025) https://doi.org/10.1101/2025.10.09.25337646 https://www.medrxiv.org/content/early/2025/10/13/2025.10.09.25337646.full.pdf

[2] Chaffin, M., Papangeli, I., Simonson, B., Akkad, A.-D., Hill, M.C., Arduini, A., Fleming, S.J., Melanson, M., Hayat, S., Kost-Alimova, M., Atwa, O., Ye, J., Bedi, K.C., Nahrendorf, M., Kaushik, V.K., Stegmann, C.M., Margulies, K.B., Tucker, N.R., Ellinor, P.T.: Single-nucleus profiling of human dilated and hypertrophic cardiomyopathy. Nature **608**(7921), 174–180 (2022) https://doi.org/10.1038/s41586-022-04817-8

[3] Kuppe, C., Ramirez Flores, R.O., Li, Z., Hayat, S., Levinson, R.T., Liao, X., Hannani, M.T., Tanevski, J., Wünnemann, F., Nagai, J.S., Halder, M., Schumacher, D., Menzel, S., Schäfer, G., Hoeft, K., Cheng, M., Ziegler, S., Zhang, X., Peisker, F., Kaesler, N., Saritas, T., Xu, Y., Kassner, A., Gummert, J., Morshuis, M., Amrute, J., Veltrop, R.J.A., Boor, P., Klingel, K., Van Laake, L.W., Vink, A., Hoogenboezem, R.M., Bindels, E.M.J., Schurgers, L., Sattler, S., Schapiro, D., Schneider, R.K., Lavine, K., Milting, H., Costa, I.G., Saez-Rodriguez, J., Kramann, R.: Spatial multi-omic map of human myocardial infarction. Nature **608**(7924), 766–777 (2022) https://doi.org/10.1038/s41586-022-05060-x

[4] Litviňuková, M., Talavera-López, C., Maatz, H., Reichart, D., Worth, C.L., Lindberg, E.L., Kanda, M., Polanski, K., Heinig, M., Lee, M., Nadelmann, E.R., Roberts, K., Tuck, L., Fasouli, E.S., DeLaughter, D.M., McDonough, B., Wakimoto, H., Gorham, J.M., Samari, S., Mahbubani, K.T., Saeb-Parsy, K., Patone, G., Boyle, J.J., Zhang, H., Zhang, H., Viveiros, A., Oudit, G.Y., Bayraktar, O.A., Seidman, J.G., Seidman, C.E., Noseda, M., Hubner, N., Teichmann, S.A.: Cells of the adult human heart. Nature **588**(7838), 466–472 (2020) https://doi.org/10.1038/s41586-020-2797-4

[5] Reichart, D., Lindberg, E.L., Maatz, H., Miranda, A.M.A., Viveiros, A., Shvetsov, N., Gärtner, A., Nadelmann, E.R., Lee, M., Kanemaru, K., Ruiz-Orera, J., Strohmenger, V., DeLaughter, D.M., Patone, G., Zhang, H., Woehler, A., Lippert, C., Kim, Y., Adami, E., Gorham, J.M., Barnett, S.N., Brown, K., Buchan, R.J., Chowdhury, R.A., Constantinou, C., Cranley, J., Felkin, L.E., Fox, H., Ghauri, A., Gummert, J., Kanda, M., Li, R., Mach, L., McDonough, B., Samari, S., Shahriaran, F., Yapp, C., Stanasiuk, C., Theotokis, P.I., Theis, F.J., van den Bogaerdt, A., Wakimoto, H., Ware, J.S., Worth, C.L., Barton, P.J.R., Lee, Y.-A., Teichmann, S.A., Milting, H., Noseda, M., Oudit, G.Y., Heinig, M., Seidman, J.G., Hubner, N., Seidman, C.E.: Pathogenic variants damage cell composition and single cell transcription in cardiomyopathies. Science (New York, N.Y.) **377**(6606), 1984 (2022) https://doi.org/10.1126/science.abo1984

[6] Sikkema, L., Ramírez-Suástegui, C., Strobl, D.C., Gillett, T.E., Zappia, L., Madissoon,

E., Markov, N.S., Zaragosi, L.-E., Ji, Y., Ansari, M., Arguel, M.-J., Apperloo, L., Banchero, M., Bécavin, C., Berg, M., Chichelnitskiy, E., Chung, M.-i., Collin, A., Gay, A.C.A., Gote-Schniering, J., Hooshiar Kashani, B., Inecik, K., Jain, M., Kapellos, T.S., Kole, T.M., Leroy, S., Mayr, C.H., Oliver, A.J., von Papen, M., Peter, L., Taylor, C.J., Walzthoeni, T., Xu, C., Bui, L.T., De Donno, C., Dony, L., Faiz, A., Guo, M., Gutierrez, A.J., Heumos, L., Huang, N., Ibarra, I.L., Jackson, N.D., Kadur Lakshminarasimha Murthy, P., Lotfollahi, M., Tabib, T., Talavera-López, C., Travaglini, K.J., Wilbrey-Clark, A., Worlock, K.B., Yoshida, M., van den Berge, M., Bossé, Y., Desai, T.J., Eickelberg, O., Kaminski, N., Krasnow, M.A., Lafyatis, R., Nikolic, M.Z., Powell, J.E., Rajagopal, J., Rojas, M., Rozenblatt-Rosen, O., Seibold, M.A., Sheppard, D., Shepherd, D.P., Sin, D.D., Timens, W., Tsankov, A.M., Whitsett, J., Xu, Y., Banovich, N.E., Barbry, P., Duong, T.E., Falk, C.S., Meyer, K.B., Kropski, J.A., Pe'er, D., Schiller, H.B., Tata, P.R., Schultze, J.L., Teichmann, S.A., Misharin, A.V., Nawijn, M.C., Luecken, M.D., Theis, F.J.: An integrated cell atlas of the lung in health and disease. Nature Medicine **29**(6), 1563–1577 (2023) https://doi.org/10.1038/s41591-023-02327-2

[7] Habermann, A.C., Gutierrez, A.J., Bui, L.T., Yahn, S.L., Winters, N.I., Calvi, C.L., Peter, L., Chung, M.-I., Taylor, C.J., Jetter, C., Raju, L., Roberson, J., Ding, G., Wood, L., Sucre, J.M.S., Richmond, B.W., Serezani, A.P., McDonnell, W.J., Mallal, S.B., Bacchetta, M.J., Loyd, J.E., Shaver, C.M., Ware, L.B., Bremner, R., Walia, R., Blackwell, T.S., Banovich, N.E., Kropski, J.A.: Single-cell RNA sequencing reveals profibrotic roles of distinct epithelial and mesenchymal lineages in pulmonary fibrosis. Science Advances **6**(28), 1972 (2020) https://doi.org/10.1126/sciadv.aba1972

[8] Travaglini, K.J., Nabhan, A.N., Penland, L., Sinha, R., Gillich, A., Sit, R.V., Chang, S., Conley, S.D., Mori, Y., Seita, J., Berry, G.J., Shrager, J.B., Metzger, R.J., Kuo, C.S., Neff, N., Weissman, I.L., Quake, S.R., Krasnow, M.A.: A molecular cell atlas of the human lung from single-cell RNA sequencing. Nature **587**(7835), 619–625 (2020) https://doi.org/10.1038/s41586-020-2922-4

[9] Grant, R.A., Morales-Nebreda, L., Markov, N.S., Swaminathan, S., Querrey, M., Guzman, E.R., Abbott, D.A., Donnelly, H.K., Donayre, A., Goldberg, I.A., Klug, Z.M., Borkowski, N., Lu, Z., Kihshen, H., Politanska, Y., Sichizya, L., Kang, M., Shilatifard, A., Qi, C., Lomasney, J.W., Argento, A.C., Kruser, J.M., Malsin, E.S., Pickens, C.O., Smith, S.B., Walter, J.M., Pawlowski, A.E., Schneider, D., Nannapaneni, P., Abdala-Valencia, H., Bharat, A., Gottardi, C.J., Budinger, G.R.S., Misharin, A.V., Singer, B.D., Wunderink, R.G.: Circuits between infected macrophages and T cells in SARS-CoV-2 pneumonia. Nature **590**(7847), 635–641 (2021) https://doi.org/10.1038/s41586-020-03148-w . Publisher: Nature Publishing Group. Accessed 2025-01-11

[10] Misharin, A.V., Budinger, G.R.S.: Targeting the Myofibroblast in Pulmonary Fibrosis. American Journal of Respiratory and Critical Care Medicine **198**(7), 834–835 (2018) https://doi.org/10.1164/rccm.201806-1037ED

[11] Reed, A.D., Pensa, S., Steif, A., Stenning, J., Kunz, D.J., Porter, L.J., Hua, K., He, P., Twigger, A.-J., Siu, A.J.Q., Kania, K., Barrow-McGee, R., Goulding, I., Gomm, J.J., Speirs, V.,

Jones, J.L., Marioni, J.C., Khaled, W.T.: A single-cell atlas enables mapping of homeostatic cellular shifts in the adult human breast. Nature Genetics **56**(4), 652–662 (2024) https://doi.org/10.1038/s41588-024-01688-9

[12] Murrow, L.M., Weber, R.J., Caruso, J.A., McGinnis, C.S., Phong, K., Gascard, P., Rabadam, G., Borowsky, A.D., Desai, T.A., Thomson, M., Tlsty, T., Gartner, Z.J.: Mapping hormone-regulated cell-cell interaction networks in the human breast at single-cell resolution. Cell Systems **13**(8), 644–6648 (2022) https://doi.org/10.1016/j.cels.2022.06.005

[13] Nee, K., Ma, D., Nguyen, Q.H., Pein, M., Pervolarakis, N., Insua-Rodríguez, J., Gong, Y., Hernandez, G., Alshetaiwi, H., Williams, J., Rauf, M., Dave, K.R., Boyapati, K., Hasnain, A., Calderon, C., Markaryan, A., Edwards, R., Lin, E., Parajuli, R., Zhou, P., Nie, Q., Shalabi, S., LaBarge, M.A., Kessenbrock, K.: Preneoplastic stromal cells promote BRCA1-mediated breast tumorigenesis. Nature Genetics **55**(4), 595–606 (2023) https://doi.org/10.1038/s41588-023-01298-x

[14] Pal, B., Chen, Y., Vaillant, F., Capaldo, B.D., Joyce, R., Song, X., Bryant, V.L., Penington, J.S., Di Stefano, L., Tubau Ribera, N., Wilcox, S., Mann, G.B., kConFab, Papenfuss, A.T., Lindeman, G.J., Smyth, G.K., Visvader, J.E.: A single-cell RNA expression atlas of normal, preneoplastic and tumorigenic states in the human breast. The EMBO Journal **40**(11), 107333 (2021) https://doi.org/10.15252/embj.2020107333

[15] Twigger, A.-J., Engelbrecht, L.K., Bach, K., Schultz-Pernice, I., Pensa, S., Stenning, J., Petricca, S., Scheel, C.H., Khaled, W.T.: Transcriptional changes in the mammary gland during lactation revealed by single cell sequencing of cells from human milk. Nature Communications **13**(1), 562 (2022) https://doi.org/10.1038/s41467-021-27895-0
